# Methylation of cell-free circulating DNA in the diagnosis of cancer

**DOI:** 10.3389/fmolb.2015.00013

**Published:** 2015-04-22

**Authors:** Kristina Warton, Goli Samimi

**Affiliations:** Garvan Institute of Medical Research, The Kinghorn Cancer Centre and St Vincent's Clinical School, University of New South WalesSydney, NSW, Australia

**Keywords:** cancer, early detection, diagnosis, circulating DNA, plasma DNA, methylation

## Abstract

A range of molecular alterations found in tumor cells, such as DNA mutations and DNA methylation, is reflected in cell-free circulating DNA (circDNA) released from the tumor into the blood, thereby making circDNA an ideal candidate for the basis of a blood-based cancer diagnosis test. In many cancer types, mutations driving tumor development and progression are present in a wide range of oncogenes and tumor suppressor genes. However, even when a gene is consistently mutated in a particular cancer, the mutations can be spread over very large regions of its sequence, making evaluation difficult. This diversity of sequence changes in tumor DNA presents a challenge for the development of blood tests based on DNA mutations for cancer diagnosis. Unlike mutations, DNA methylation that can be consistently measured, as it tends to occur in specific regions of the DNA called CpG islands. Since DNA methylation is reflected within circDNA, detection of tumor-specific DNA methylation in patient plasma is a feasible approach for the development of a blood-based test. Aberrant circDNA methylation has been described in most cancer types and is actively being investigated for clinical applications. A commercial blood test for colorectal cancer based on the methylation of the *SEPT9* promoter region in circDNA is under review for approval by the Federal Drug Administration (FDA) for clinical use. In this paper, we review the state of research in circDNA methylation as an application for blood-based diagnostic tests in colorectal, breast, lung, pancreatic and ovarian cancers, and we consider some of the future directions and challenges in this field. There are a number of potential circDNA biomarkers currently under investigation, and experience with *SEPT9* shows that the time to clinical translation can be relatively rapid, supporting the promise of circDNA as a biomarker.

## DNA methylation as a biomarker

One of the surprising aspects of cancer biology that emerged from The Cancer Genome Atlas (TCGA) sequencing projects was the wide diversity of mutations that give rise to cancer (Vogelstein et al., 2013). Even within a single tumor type the mutation profiles vary from patient to patient, and it's not unusual for even the most commonly altered genes to be mutated in less than half of the cases. For example, TCGA sequencing of breast cancer showed that the two most commonly mutated genes, *TP53* and *PIK3CA*, are only mutated in 37 and 36% of tumors, respectively (Cancer Genome Atlas, 2012). The mutation frequency then drops off sharply, with most of the remaining top 23 genes mutated in <5% of tumors. Even when a single gene is commonly mutated in a particular cancer type, the sequence changes can be spread over large stretches of DNA. For example, *TP53* is one of the most consistently altered genes in high grade serous ovarian cancer (HGSOC), with around 95% of tumors harboring a mutation (Ahmed et al., 2010; Cancer Genome Atlas Research Network, 2011). However, as predicted for a tumor suppressor gene (Vogelstein et al., 2013), the mutations show minimal clustering and are spread over several exons (Hollstein et al., 1991; Cancer Genome Atlas Research Network, 2011), which span nearly 20 kilobases of sequence. The TCGA ovarian cancer sequencing project identified seven other significantly mutated genes, but these were only present in 2–6% of samples (Cancer Genome Atlas Research Network, 2011). This diversity of mutations provides a challenge for the development of cancer diagnosis tests based on DNA sequence changes, as very large proportions of the genome would need to be interrogated to provide a test of adequate sensitivity (Schmidt and Diehl, 2007).

The variability of cancer mutation profiles contrasts with the stability of CpG island methylation changes. The *14-3-3 sigma* promoter has been found to be methylated in 96% of breast carcinomas, and unmethylated in the breast epithelium of individuals without cancer (Umbricht et al., 2001). While not a good candidate biomarker for a breast cancer blood test, since *14-3-3 sigma* is also heavily methylated in leukocytes (Umbricht et al., 2001), this level of methylation underlies the homogeneity of certain DNA methylation changes as compared with mutations. Along the same lines, the *HOXA9* promoter and *EN1* promoter were found to be methylated in 95 and 80% of HGSOC ovarian cancers, respectively (Montavon et al., 2012). Given the greater consistency of DNA methylation changes in cancer compared to mutations, methylation is a promising target for biomarker development.

## Cell-free circulating plasma DNA

Cell-free circulating plasma DNA (circDNA) is DNA found in blood plasma which is not associated with any cell fraction. circDNA is generally shed from normal cells, including leukocytes. In individuals with cancer a proportion of circDNA is derived from tumor cells, and not only contains the same mutations as tumor cells, but also the same methylation pattern (Schwarzenbach et al., 2011). Furthermore, studies have demonstrated that circDNA can be detected in most patients harboring solid tumors with advanced disease, as well as in a lower fraction of patients with localized disease (Bettegowda et al., 2014). Thus, tumor-specific methylation in circDNA is a potential target for the development of non-invasive, blood-based assays for cancer diagnosis (Supplementary Table 1).

circDNA has been extracted from both plasma and serum. Serum typically yields higher amounts of DNA (Lo et al., 1998; Lee et al., 2001; Lui et al., 2002; Jung et al., 2003; Warton et al., 2014); however, there is evidence that the additional DNA seen in serum is in fact derived from leukocytes which lyse during serum processing, rather than a reflection of greater amounts of circDNA (Lee et al., 2001; Warton et al., 2014). Supporting this notion, serum from female blood spiked with male-derived leukocytes prior to isolation contains Y-chromosome sequences, indicating that leukocytes are subject to lysis during the clotting process (Lee et al., 2001). As an alternate hypothesis, the higher circDNA concentration in serum has been postulated to be due to chemical differences between plasma and serum which render serum more amenable to DNA extraction. However, levels of serum circDNA have been shown to rise with even short (2–8 h) increases in clotting times, during which serum chemistry is not likely to change (Jung et al., 2003; Warton et al., 2014). Instead the observation of increased yield with prolonged clotting is consistent with ongoing leukocyte lysis. Serum may still prove a valuable source of biomarkers if the biomarker chosen is not compromised by an increased level of background leukocyte DNA, or even if it itself is derived from lysed leukocytes. However, to avoid clouding the issue we have chosen to focus this review on plasma, which we believe is less likely to be impacted by sample processing artifacts.

## Colorectal cancer

Colorectal cancer is the 3rd most common diagnosed cancer and cause of cancer-related death in both men and women (Siegel et al., 2014). Tests for the presence of colorectal cancer in the form of sigmoidoscopy/colonoscopy and the fecal occult blood test (FOBT) have existed for over 50 years and are an illustrative example of the potential of screening to reduce cancer mortality, with several large, long-term follow-up studies demonstrating the benefit of screening. The Minnesota Colon Cancer Control Study showed a relative risk of 0.68 (95%CI 0.56–0.82) among participants randomized to annual FOBT screening compared to the control group over 30 years of follow-up (Shaukat et al., 2013). The Nurses' Health Study (Nishihara et al., 2013) and the Nottingham trial (Scholefield et al., 2012) also showed reductions in colorectal cancer mortality following colonoscopy/sigmoidoscopy and FOBT screening, respectively; however, screening compliance in the Nottingham trial was only around 60%, highlighting a potential area for improvement for existing tests.

New screening tests for colorectal cancer seek improvements in sensitivity and specificity of the diagnosis, but also alternative formats to fecal screening or colonoscopy, which have been shown to have low uptake in a population screening setting (Scholefield et al., 2012). The *septin 9* (*SEPT9*) gene promoter region was initially identified as being differentially methylated in a discovery project comparing colorectal tumors, healthy adjacent tissues, peripheral blood lymphocytes, and other non-colonic, non-pathologic control tissues which could hypothetically contribute DNA to plasma (Lofton-Day et al., 2008). As the biomarker was intended specifically for development into a blood test, there was a stringent requirement at the discovery phase that it shows minimal or absent levels of methylation in leukocytes.

Following identification as a potential biomarker, *SEPT9* was evaluated in retrospective trials comparing plasma methylation in people diagnosed with colorectal cancer and healthy controls (Grutzmann et al., 2008; Devos et al., 2009; Warren et al., 2011). When these gave promising results, ranging between 72–90% for sensitivity and 88–90% for specificity, a large scale, multicentre prospective study, the PRESEPT trial, was undertaken (Church et al., 2014). This trial enrolled over 7900 participants from among patients scheduled for colonoscopy, 53 of whom were on examination found to have colorectal cancer. In the trial the *SEPT9* methylation assay showed a disappointing 48.2% sensitivity for detecting pre-clinical colorectal cancer, with a 91.5% specificity. A suggested reason why this prospective trial did not perform as well as the retrospective study is that the retrospectively collected samples were from cases diagnosed symptomatically rather than in a screening setting, and these may have differed from asymptomatic screen-detected cases. However, in the course of the PRESEPT trial a simple technical change to the assay, designating a patient sample as positive when 1 of 3 rather than 1 of 2 PCR reactions showed methylated *SEPT9* amplification, was identified as giving better sensitivity and incorporated into the trial. This resulted in a sensitivity 63.9% and a specificity of 88.4% (Warren et al., 2011; Church et al., 2014). The effect of this modification is yet to be prospectively evaluated in a large scale, multicentre trial; however, a de-novo re-testing of the PRESEPT participant samples appears encouraging (Potter et al., 2014).

While other diagnostic modalities may perform better than the methylated *SEPT9* test at the assay level (Ahlquist et al., 2012; Ladabaum et al., 2013), low testing uptake has been consistently identified as a problem in colorectal cancer screening, and insofar as the *SEPT9* test can overcome this it is adequately sensitive to provide patient benefit as well as cost effective (Ladabaum et al., 2013). The *SEPT9* test also has potential for further technical improvement, for example, the PCR reaction on which the test is based appears sensitive to blood derived inhibitors which are responsible for a proportion of the false negative results (Grutzmann et al., 2008) and, despite some effort in this direction, these inhibitors are yet to be identified (Potter et al., 2014). Overall, the *SEPT9* test is a very encouraging example of bringing a methylation-based blood biomarker from the laboratory to the clinic.

Other methylation-based plasma biomarkers that differentiate control blood samples and samples from patients with colorectal cancer have been identified, both through whole genome screening (Lange et al., 2012; Pedersen et al., 2014; Takane et al., 2014) as well as through candidate gene testing (Lee et al., 2009; Cassinotti et al., 2011; Danese et al., 2013; Pack et al., 2013; Melotte et al., 2015) approaches. These include the promoters of genes which have a well-established role in cancer progression such as *RASSF1A, APC* and *E-cadherin* (Cassinotti et al., 2011; Pack et al., 2013), as well as completely novel sequences such as *CAHM*, a long non-coding RNA gene (Pedersen et al., 2014). In these initial studies some of the methylated sequences showed sensitivity and specificity that compare well to early results obtained with *SEPT9*; for example, the promoter region of *THBD* was able to differentiate colorectal cancer and control blood samples with a sensitivity of 71% and a specificity of 80% (Lange et al., 2012). However, the new markers described have yet to be evaluated in independent studies, either retrospectively or prospectively, which would give a clearer idea of their potential clinical utility.

## Breast cancer

As with colorectal cancer, non-blood-based screening tests for breast cancer are already in clinical use. Mammography and ultrasound can reveal suspicious tissue lesions, and these can be followed up by fine needle biopsy if necessary. However, the sensitivity and specificity of these tests are only moderate and vary greatly (Drukteinis et al., 2013), and patient benefits of population screening can be difficult to detect, even with very large, well-powered clinical trials (Independent UK Panel on Breast Cancer Screening, 2012). Concerns regarding existing tests are that over-diagnosis leads to unnecessary surgery on lesions that would not have progressed further, while many malignant tumors remain undetected (Independent UK Panel on Breast Cancer Screening, 2012). Hence the hope is that a blood test would improve on the sensitivity and specificity of current screening.

A number of potential methylation biomarkers that differentiate control plasma and plasma from patients with breast cancer have been found, mostly using the candidate gene approach (Hoque et al., 2006; Skvortsova et al., 2006; Ng et al., 2011; Radpour et al., 2011; Chimonidou et al., 2013a,b; Guerrero-Preston et al., 2014). As such, the regions identified lie in the promoters of genes already well-defined as having a role in cancer, for example *RASSF1A* (Hoque et al., 2006; Skvortsova et al., 2006), *APC* (Hoque et al., 2006; Radpour et al., 2011) and *SOX17* (Chimonidou et al., 2013a). Notably, *CST6* has been identified by two independent groups as being differentially methylated between breast cancer and control plasma samples (Radpour et al., 2011; Chimonidou et al., 2013b). Chimonidou et al. (2013b) used bisulphite conversion and methylation-specific PCR of circDNA to show that the *CST6* promoter was methylated in two separate cohorts of breast cancer patients, with methylation found in 14/73 patients (19.2%) in the first cohort, and in 49/123 patients (39.8%) in the second cohort, while none of the 37 healthy individuals tested showed methylation. One caveat of this study is that the plasma samples were collected 2–4 weeks after surgery, and a decrease in tumor-derived circDNA following tumor removal is well-documented (Diehl et al., 2008; Li et al., 2009).

The highest sensitivity and specificity for detection was obtained using an 8-gene biomarker panel (that included *CST6*), which correctly identified 91.7% of cancer samples, and 90% of cancer negative samples in a retrospective cohort consisting of 36 patients mostly with early stage breast cancer, and 30 healthy controls (Radpour et al., 2011). However, this study also showed an identifiable level of *CST6* methylation in the control samples (Radpour et al., 2011).

To our knowledge, to date there have only been reports presenting the first description of potential methylation biomarker(s) in breast cancer using relatively small sample numbers, and these have not as yet been followed up by independent larger studies, either prospective or retrospective. It is also not known whether the most appropriate biomarkers differ by breast cancer subtype. So far biomarker discovery studies have generally been performed on mixed cohorts of ER-positive and ER-negative patients, potentially further decreasing the power of the trials.

## Lung cancer

Despite recent decreases in smoking in developed nations, the lag time between carcinogen exposure and the development of tumors means that lung cancer continues to be responsible for the biggest fraction of cancer mortality among both men and women (Siegel et al., 2014). Methylated *CDKN2A* (often designated as *p16*) was an early focus of the search for a plasma diagnostic biomarker for lung cancer; however, while the early studies identified *CDKN2A* promoter methylation in the plasma of lung cancer patients, they either had very small numbers of healthy controls (Bearzatto et al., 2002), or they did not include a cancer-free control group in their analysis (Kurakawa et al., 2001; An et al., 2002; Ng et al., 2002), extrapolating instead from the lack of *CDKN2A* methylation in plasma of patients whose tumors were *CDKN2A* methylation free. Subsequent studies found *CDKN2A* methylation in the plasma of 7/74 (9%), 3/33 (9%), and 4/50 (8%) of cancer-free controls (Belinsky et al., 2005; Hsu et al., 2007; Zhang et al., 2011), while methylation among patients with cancer included values of 25/110 (22%), 21/55 (38%), and 61% sensitivity (Wang et al., 2006; Hsu et al., 2007; Zhang et al., 2011). These numbers suggest that if methylated plasma *CDKN2A* is to be useful in detecting lung cancer, it will likely be as part of a biomarker panel rather than as a single gene.

Other promoters which were found by independent studies to be differentially methylated in circDNA between patients with cancer and cancer free controls include *APC* (Usadel et al., 2002; Rykova et al., 2004; Zhang et al., 2011), *RASSF1A* (Rykova et al., 2004; Belinsky et al., 2005; Wang et al., 2006; Hsu et al., 2007; Ponomaryova et al., 2011; Zhang et al., 2011), *RARb* (Rykova et al., 2004; Wang et al., 2006; Hsu et al., 2007; Ostrow et al., 2010; Ponomaryova et al., 2013), and *CDH13* (Rykova et al., 2004; Wang et al., 2006; Hsu et al., 2007; Zhang et al., 2011); however, the sensitivities were generally low. A relatively well-powered, retrospective study by researchers from the diagnostic firm Theracode identified *SHOX2* as a potential biomarker (Kneip et al., 2011). An initial training set of 20 lung cancer cases and 20 controls was used to establish an assay cutoff value, and then a total of 371 cancer and control samples were tested and found a sensitivity of 60% (CI 53–67%) and a specificity of 90% (CI 84–94%) (Kneip et al., 2011). Finally, the *SEPT9* methylation test originally developed for plasma detection of colorectal cancer has been applied to lung cancer, and correctly identified 31/70 (44%) of lung cancer samples, while only giving a positive signal in 4/100 controls (Powrozek et al., 2014).

The development of a lung cancer biomarker presents particular problems in the choice of an appropriate control group. In the majority of cases lung cancer is linked to smoking, and smoking itself leads to gene methylation changes (Ostrow et al., 2013). Hence smokers with no evidence of lung cancer are a good control group, as this approach would avoid selection of methylation markers which simply identify people who smoke. However, some of the potential circDNA methylation biomarkers examined have been found to have differences in specificity, i.e., the ability to correctly identify negative samples, between smoker and non-smoker controls (Ostrow et al., 2010). Without lengthy follow-up of the negative control subjects, it is not clear whether these signals in the smoker group are false positives, or detection of early events in the initiation of lung cancer (Ostrow et al., 2010).

## Pancreatic cancer

Pancreatic cancer is one of the most lethal cancers in men and women (Siegel et al., 2014). Late detection of tumors due to few symptoms at early stages means that, by the time the cancer has been diagnosed, it has spread such that it cannot be surgically removed. As with other cancers, earlier detection improves patient outcomes. Compared to patients with advanced tumors, patients with tumors ≤1 cm at surgery appear to do relatively well, with >70% 5-year survival (Ishikawa et al., 1999). The small size of the target lesion is cited as one of the reasons for the difficulty of early pancreatic cancer detection, as tumors of this size are considered unlikely to shed large amounts of DNA into the blood (Lennon et al., 2014).

A recent study by Nones et al. (2014), as part of the Australian Pancreatic Cancer Genome Initiative (APGI), found high levels of aberrant methylation in crucial signaling pathways, suggesting its feasibility as a biomarker for disease. With regards to plasma methylated biomarker discovery, the pancreatic cancer field is still in early stages, and the number of studies carried out to date is small. One of the first studies identified *PENK* and *CDKN2A* methylation in the plasma of 21.4 and 45.4% of patients with localized pancreatic cancer, however, this study did not include a cancer-free control group (Jiao et al., 2007). Using a panel of 6 candidate genes, *UCHL1, NPTX2, SARP2, ppENK, CDKN2A* and *RASSF1A*, Park showed that while all gene promoters were differentially methylated between patients with pancreatic cancer and healthy controls, only one gene promoter, *CDKN2A*, was differentially methylated between patients with pancreatic cancer and those with chronic pancreatitis, a known risk factor for pancreatic cancer (Park et al., 2012).

One discovery technique involves a microarray test panel of 56 frequently methylated genes that is used to measure the quantity of methylated target sequence following digestion with the methylation sensitive endonuclease Hin6I, and PCR amplification of undigested (i.e., methylated) fragments (MethDet56) (Melnikov et al., 2009b). This technique has been used to identify promoters that are hypomethylated in the plasma of pancreatic cancer patients relative to controls. *CCND2, SOCS1, THBS, PLAU*, and *VHL* were identified as being unmethylated in cancer samples relative to healthy controls (Melnikov et al., 2009b). However, the application of a biomarker based on decreased methylation is problematic. While shedding of hypomethylated tumor DNA into the bloodstream may lead to a decrease in the circDNA methylation signal, tumor DNA would have to form a large proportion of circDNA for this decrease to be detectable. It is unlikely that the tiny amounts of tumor DNA introduced into the bloodstream from early stage cancers would result in a detectable decrease in DNA methylation. On the other hand, lack of detectable methylation is potentially useful in distinguishing cancer from conditions which present with similar symptoms. The MethDet56 method described above was used to differentiate between healthy controls, pancreatic cancer patients and patients with chronic pancreatitis, with sequences that were methylated in chronic pancreatitis but unmethylated in pancreatic cancer contributing to the specificity of the biomarker (Liggett et al., 2010).

## Ovarian cancer

Symptoms of ovarian cancer are vague and non-specific, leading to late diagnosis which in turn results in high patient mortality. Several large scale efforts have been undertaken to determine the efficacy of ovarian cancer screening using different configurations of CA125 measurement and ultrasound imaging, including the US PLCO Cancer Screening Study (Buys et al., 2011) and the UKCTOCS study (Menon et al., 2009). However, to date, these screening practices have not shown an improvement in mortality (reviewed in Menon et al., 2014). There is scope for improvement in the biomarkers used to detect ovarian cancer. CA125, one of the cornerstones of ovarian cancer screening trials, was first reported as being elevated in ovarian cancer over 30 years ago (Bast et al., 1983), that is, even before the invention of PCR. Since that time, molecular biology has been transformed beyond recognition, and it seems reasonable to hope that new techniques, vastly more powerful than anything envisaged 3 decades ago, will enable the identification of biomarkers that improve on the sensitivity and specificity of CA125.

While many studies have reported aberrent methylation in ovarian cancer (reviewed in Gloss and Samimi, 2014), there are relatively few reports of ovarian cancer plasma methylation biomarkers. Ibanez De Caceres et al. (2004) found methylation of *RASSF1A* and *BRCA1* promoters in plasma or serum in 25/50 (50%) and 9/50 (18%) of ovarian cancer samples, respectively, with neither promoter methylated in any of 20 controls. The number of negative control samples was insufficient to allow precise conclusions about specificity, while other studies have found both *RASSF1A* and *BRCA1* to be methylated in small numbers of controls (Belinsky et al., 2005; Hsu et al., 2007; Radpour et al., 2011; Zhang et al., 2011); however, assay differences make direct comparison across these studies difficult. The MethDet56 method described above has been applied to ovarian cancer, and several genes were found informative in identifying ovarian cancer samples (Melnikov et al., 2009a; Liggett et al., 2011). In a cohort of 30 patients with ovarian cancer, 30 patients with benign ovarian disease and 30 healthy controls, methylation of *RASSF1A, CACLA*, and *EP300* combined differentiated between patients with ovarian cancer and healthy controls with 90% sensitivity and 87% specificity, while methylation of *RASSF1A* and *PGR* differentiated between patients with ovarian cancer and patients with benign ovarian disease with 80.0% sensitivity and 73.3% specificity (Liggett et al., 2011).

Because ovarian cancer is relatively rare, and diagnosed late, plasma samples from early stage cases that could be used to validate useful biomarker(s) are rare. Serum for CA125 measurement is usually collected from ovarian cancer patients on diagnosis, and hence serum is more often readily available for biomarker studies in ovarian cancer. A number of studies identifying serum methylation ovarian cancer biomarkers have been published (Dong et al., 2012; Zhang et al., 2013; Zhou et al., 2014), however, a detailed description of these is beyond the scope of this review.

## Challenges in working with methylated circDNA

Methylated circDNA presents a challenging substrate to work with, largely because circDNA is dilute, with concentrations as low as < 10 ng per mL of plasma in healthy subjects (El Messaoudi et al., 2013; Warton et al., 2014). The methylated component is an even smaller subfraction of this amount. Thus, many of the technical issues relate simply to limited quantities of starting material, a common problem for researchers working with clinical samples. This can be partly addressed through the development of DNA purification methods which accommodate larger volumes of plasma input (Keeley et al., 2013; Warton et al., 2014); however, care needs to be taken that this doesn't compound problems stemming from blood-derived PCR inhibitors (Grutzmann et al., 2008; Schrader et al., 2012). Fortunately, even for low volumes of plasma starting material, ongoing technical developments mean that it is possible to analyze ever smaller amounts of DNA.

As noted above, the size of early stage tumors may limit the amount of DNA that is shed into the blood. However, there are many unknown parameters in estimating how much DNA a tumor is likely to release into the blood stream, starting from the number of cells per cubic centimeter of tumor tissue, which has been postulated to range from 1 × 10^8^ to 1 × 10^9^ per cm^3^ depending on tumor cell size and the proportion of stromal components (Del Monte, 2009), to the rate and mechanism of DNA release, both of which very little is known about at this stage. A 1 cm diameter tumor, containing about half a billion cells, with only 0.01% genome equivalents in the circulation would present as 17–20 tumor genomes, or 34–40 copies of a given sequence per mL of plasma, assuming average human plasma volume to be 3 L for men and 2.5 L for women. With reported optimal limits of methylation detection being 1–2 copies of DNA sequence per assay (Pedersen et al., 2014), and with the option of detecting targets concentrated from several mL of plasma in a single multiplexed assay once issues of blood-derived inhibitors are resolved, it is conceivable that methylated DNA from tumors small enough to be surgically removed could be detected. However, for assays that detect target molecules with this very high sensitivity to be effective, it is important that the chosen targets are not methylated even at low levels in control blood samples, as the specificity of the test would be compromised.

Another challenge in circDNA studies is that circDNA is fragmented. Most of the DNA present in plasma occurs as fragments around 180 bases and 360 bases in size. This corresponds to the smallest two bands of the apoptotic DNA ladder, and reflects the likely apoptotic origin of the DNA (Jahr et al., 2001). A technical consequence of this is that DNA purification methods that perform well for intact genomic DNA are not efficient at extracting circDNA (Devonshire et al., 2014), leading to further DNA losses (and also inconsistencies in circDNA concentrations measured by different labs).

The collection and processing of clinical samples for the discovery and validation of plasma biomarkers also needs to be carefully considered. Blood in EDTA tubes can only be stored for a limited amount of time before leukocytes begin to lyse and contribute their DNA to the plasma DNA fraction (Jung et al., 2003; El Messaoudi et al., 2013; Warton et al., 2014). At the very least, same day processing of blood samples is required so that the circDNA does not become contaminated with genomic leukocyte DNA. In clinical settings where patient samples are collected one at a time, sample collection and processing can become a major enterprise. Furthermore, determining the specificity of a biomarker, that is, its ability to correctly identify negative samples, requires large numbers of cancer-free controls, ideally age-matched and collected within the same setting and in the same way as the cases. If control samples are collected at different centers than the cancer samples, standardized protocols are required so that variations in sample handling between centers do not introduce artifacts (Gormally et al., 2004). Problems can also arise from discovery and validation strategies that compare samples from patients with cancer to samples from healthy individuals, insofar as in addition to cancer specific biomarkers, the patient samples are also likely to show changes due simply to the presence of inflammation. The resulting biomarker may not be able to differentiate between cancer and other less serious conditions with an inflammation component. These considerations are not specific to methylated DNA biomarkers and apply to biomarker discovery and validation generally.

Developing a diagnostic test for rare cancers such as pancreatic or ovarian cancer is more technically difficult than developing a test for common cancers such as breast and colorectal cancer. This is because the assay adopted for rare cancers must have an extremely low false positive rate if the test is to be clinically useful. A 100% sensitive test with 95% specificity (i.e., 5% false positive rate), if targeted at colorectal cancer, would return on average 1 correct cancer diagnosis and 8 false positive results after screening 160 patients, whereas the same test applied to ovarian cancer screening would return on average 1 correctly identified ovarian cancer diagnosis and 125 false positive results after screening 2500 patients, assuming the prevalence of colorectal and ovarian cancer in the screened population to be ~1/160 and ~1/2500, respectively (Rauh-Hain et al., 2011; Church et al., 2014). Furthermore, colorectal and breast cancer are located at sites where a confirmatory diagnosis can be obtained by the relatively non-invasive procedures of colonoscopy and fine needle biopsy, respectively, whereas pancreatic cancer and ovarian cancer can require surgery for a final diagnosis to be made, thus a false positive test result has much more serious consequences for patients. The problems associated with a requirement for very high sensitivity can be to some extent overcome by targeting the screening test to at-risk populations with higher prevalence of the cancer, e.g., *BRCA1/2* mutation carriers for ovarian cancer screening.

## Future directions

Technology has been evolving rapidly and much of the progress in molecular biology involves techniques that can be directly applied to DNA biomarker discovery and validation. Firstly, at the discovery stage, it has become possible to interrogate ever greater portions of the genome. From studies of one or several candidate promoters, to microarrays that measure methylation first at tens of thousands (Hill et al., 2011), then at hundreds of thousands (Pan et al., 2012) of CpG sites, and finally to whole genome sequencing that can be applied to bisulphite converted DNA (Hovestadt et al., 2014) or to the captured methylated fraction of a DNA sample (Nair et al., 2011; Warton et al., 2014), the scope to identify differentially methylated regions is increasing. The broader choice of candidates makes it more likely that a biomarker, or panel of biomarkers, will be found that is well able to differentiate between controls and cancer patient samples. For rare cancers, it is particularly important that the biomarker(s) are not methylated in cancer-free individuals. While past studies have often undertaken biomarker discovery by comparing tumors with healthy adjacent matched tissue, the methylation state of the healthy tissue of origin is not strictly speaking relevant as a negative control if the healthy tissue does not contribute DNA to the circDNA pool. A more appropriate negative control is the leukocyte population, since these are the most likely source of circDNA (Lui et al., 2002), and leukocytes have been included as negative controls in discovery studies that successfully translated to clinical biomarkers, e.g., *SEPT9* (Lofton-Day et al., 2008). Another option is to use plasma directly as the discovery substrate, with methylation of identified sequences then confirmed in corresponding tumor tissue. The advantage of this approach is that all the sources of circDNA in healthy individuals need not be identified in order for them to be included in the negative control, since if a tissue type contributes DNA to the circDNA pool, then it will be present in the tested samples. A limitation to this approach is that while the tiny amounts of circDNA in plasma can be effectively interrogated in a diagnostic assay based on PCR, they do not lend themselves to genome-wide discovery strategies that typically require larger starting inputs of DNA; however, ongoing technical improvements are likely to overcome this.

At the individual sequence level, the capacity to measure methylation is improving in sensitivity. For example, both conventional and quantitative methylation-specific PCR require that the methylated sequence is detected in a background, usually an excess, of the related unmethylated sequence. Digital PCR, in which the sample is diluted and partitioned into thousands or millions of microdroplets, amplifies and detects a single target sequence in a nanoliter reaction volume, helping to reduce competition from the non-specific product (Day et al., 2013). Another approach to eliminating competition from related sequences is to carry out the PCR on solid phase media such as polyacrylamide gel, and directly count the PCR-generated molecular colonies, each corresponding to a single starting molecule of target DNA (Chetverin and Chetverina, 2008). These emerging techniques may improve the sensitivity of PCR-based methylation assays, allowing biomarker detection in samples with less tumor DNA than is currently possible.

Experience with studies conducted to date is leading to a better appreciation of the importance of sample handling and pre-analytical processing (Gormally et al., 2004; El Messaoudi et al., 2013). Unfortunately, the blood fractionation and circDNA extraction steps are not always described in detail in published studies, which makes comparison and interpretation of results obtained by different labs difficult. Changes to sample processing based on a better understanding of the biology of circDNA may also help to improve yield. Studies from the Laktionov lab have consistently reported two pools of extracellular DNA in blood, the circDNA in plasma, and a cell surface-bound pool, which is considerably more abundant than the plasma pool (Tamkovich et al., 2008; Ponomaryova et al., 2011). The differences between these two pools of DNA need to be further clarified (Rykova et al., 2012), and any improvements in purified DNA yield would be of great benefit to the field. Another pool of cell-free plasma DNA is contained in blood exosomes, which have recently been shown to contain double-stranded DNA in addition to mRNAs and microRNAs (Thakur et al., 2014). In patients with cancer, microsomal DNA includes DNA that is derived from the tumor (Thakur et al., 2014), and hence may also be targeted in circulating tumor DNA methylation analysis. Finally, it is hoped that the emergence of personalized medicine will also boost and improve bio-banking procedures, with easier access to carefully collected and annotated cohorts of patient and control clinical samples expediting the retrospective validation step of cancer biomarker discovery.

In conclusion, a circDNA methylation biomarker for colorectal cancer is already in the clinic, and many more are under investigation. Technological developments, technical improvements, and a better understanding of circDNA biology are likely to yield targets and assays that improve on previous outcomes. There is reason to believe that these may be able to reach the sensitivity and specificity values necessary to translate into a patient survival benefit through improved detection and complete surgical removal of early stage cancers.

## Author contributions

KW and GS both contributed to the writing and editing of this manuscript.

### Conflict of interest statement

The authors declare that the research was conducted in the absence of any commercial or financial relationships that could be construed as a potential conflict of interest.
