# Supplementary material for: Methylation of cell-free circulating DNA in the diagnosis of cancer
Source: Front Mol Biosci. 2015 Apr 22;2:13. doi: 10.3389/fmolb.2015.00013 (PMC4428375; doi:10.3389/fmolb.2015.00013)
Supplement: Supplementary file 1 [file Table1.PDF]

Gene	Cancer Type	References
ANKRD18B	lung	Liu 2013;
APC	breast<br>lung<br>colerectal	Hoque 2006; Radpour 2011;<br>Usadel 2002; Rykova 2004; Zhang 2011;<br>Lee 2009; Pack 2013
BIN1	breast	Radpour 2011;
BLU	lung	Wang 2006; Hsu 2007;
BMP6	breast	Radpour 2011;
BRCA1	breast<br>ovarian	Radpour 2011;<br>Ibanez de Caceres 2004; Melnikov 2009;
C9ORF50	colorectal	Lange 2012;
CAHM	colorectal	Pedersen 2014;
CCND2	colorectal<br>pancreatic	Cassinotti 2011;<br>Melnikov 2008;
CDH1	colorectal	Pack 2011;
CDH13	lung	Wang 2006; Hsu 2007; Zhang 2011;
CDKN1A	breast	Radpour 2011;
CDKN2A	breast<br>lung<br><br><br>pancreatic	Radpour 2011; Lee 2012;<br>Kurakawa 2001; Ng 2002; An 2002; Bearzatto 2002; Liu 2003;<br>Belinsky 2005; Wang 2006; Hsu 2007; Vaissiere 2009; Zhang 2011;<br>Li 2014;<br>Li 2007;
CST6	breast	Radpour 2011: Chimonidou 2012;
DCC	lung	Ostrow 2010;
DLEC	lung	Zhang 2010; Zhang 2011;
DLEU1	lung	Balgkouranidou 2014;
EFEMP1	lung	Zhang 2011;
EFHD1	colorectal	Takane 2014;
FHIT	lung	Wang 2006; Hsu 2007;
GSTP1	breast	Hoque 2006; Radpour 2011;
HIC1	breast<br>ovarian<br>colorectal	Skvortsova 2006;<br>Melnikov 2009;<br>Cassinotti 2011;
KIF1A	lung	Ostrow 2010;
KLK10	lung	Zhang 2009; Zhang 2011;
MAL	breast	Guerrero-Preston 2014;
MDR1	colorectal	Cassinotti 2011;
MGMT	lung<br>colorectal	Belinsky 2005;<br>Lee 2009
MLH1	lung	Vaissiere 2009;
MTHFR	lung	Vaissiere 2009;
NISCH	lung	Ostrow 2010;
PAX5	ovarian<br>colorectal	Melnikov 2009;<br>Cassinotti 2011;
PENK	pancreatic	Li 2007;
PGR	ovarian	Melnikov 2009; Liggett 2011;
PLAU	pancreatic	Melnikov 2008;
PPP1R3C	colorectal	Takane 2014;

OPCML	ovarian	Zhou 2014;
RARb2	breast<br>lung	Hoque 2006; Skvortsova 2006;<br>Wang 2006; Hsu 2007; Ostrow 2010; Zhang 2011;<br>Ponomaryova 2013;
RASSF1A	breast<br>colorectal<br>lung<br><br>ovarian	Hoque 2006; Skvortsova 2006;<br>Cassinotti 2011;<br>Rykova 2004; Belinsky 2005; Wang 2006; Hsu 2007;<br>Vaissiere 2009; Zhang 2011; Ponomaryova 2013; Keely 2013;<br>Liggett 2011;
RASSF2A	colorectal	Lee 2009;
RB1	colorectal	Cassinotti 2011;
SCL19A3	breast	Ng 2011;
SEPT9	colorectal<br><br>lung	Grutzman 2008; deVos 2009; Warren 2011; Toth 2012;<br>Church 2014; Potter 2014;<br>Powrozek 2014;
SFRP1	lung	Zhang 2010; Zhang 2011
SHOX2	lung	Kneip 2011;
SHP1	lung	Vinayanuwattikun 2011; Vinayanuwattikun 2013;
SOCS2	pancreatic	Melnikov 2008;
SOX17	breast	Chimonidou 2012:
SRBC	colorectal	Cassinotti 2011;
THBD	colorectal	Lange 2012;
THBS1	ovarian<br>pancreatic	Melnikov 2009;<br>Melnikov 2008;
TIMP3	breast	Radpour 2011;
WHL	pancreatic	Melnikov 2008;
WIF1	colorectal	Lee 2009;
